# Entzündlich rheumatische Erkrankungen bei PatientInnen mit Post-COVID-Syndrom

**DOI:** 10.1007/s00393-025-01735-8

**Published:** 2025-10-14

**Authors:** N. Kippenbroek, A. Stölting, D. Schröder, M. Wetzke, C. Happle, C. Dopfer, T. Schmachtenberg, T. Witte, S. Steffens, M. Mikuteit, F. Müller, G. M. N. Behrens, A. Dopfer-Jablonka

**Affiliations:** 1 Klinik für Rheumatologie und Immunologie, Medizinische Hochschule Hannover, Carl Neuberg Str. 1, 30625 Hannover, Deutschland; 2 Institut für Allgemeinmedizin, Universitätsmedizin Göttingen, Göttingen, Deutschland; 3 Standort Hannover-Braunschweig, Deutsches Zentrum für Infektionsforschung, Braunschweig, Deutschland; 4 Klinik für Pädiatrische Pneumologie, Allergologie und Neonatologie, Medizinische Hochschule Hannover, Hannover, Deutschland; 5 Exzellenzcluster RESIST, Standort Hannover-Braunschweig, Hannover, Deutschland; 6 Lehr- und Lernforschung, Medizinische Hochschule Hannover, Hannover, Deutschland; 7 Klinik für Dermatologie und Allergologie, Medizinische Hochschule Hannover, Hannover, Deutschland

**Keywords:** Rheumatische Erkrankung, Long-COVID, ANA, Rheumatoide Arthritis, Arthritis, Rheumatic diseases, Long-COVID, Antinuclear antibodies, Rheumatoid arthritis, Arthritis

## Abstract

**Hintergrund:**

Das Post-COVID-Syndrom (PCS) beschreibt lang anhaltende Symptome nach einer SARS-CoV-2-Infektion. PCS und rheumatischen Erkrankungen, insbesondere Kollagenosen, zeigen eine hohe Schnittmenge von Symptomen und Biomarkern. Bislang existieren keine Biomarker, die PCS-PatientInnen mit und ohne rheumatischen Erkrankungen unterscheiden, und es gibt nur wenig Daten zur Prävalenz rheumatischer Erkrankungen in diesem Kollektiv in Deutschland.

**Methode:**

Basierend auf der Online-Plattform DEFEAT-Corona rekrutierten wir *n* = 80 Menschen mit PCS-Erkrankung ohne zuvor gesicherte entzündlich rheumatische Erkrankung (ERE) mit Interesse an rheumatologischer Abklärung. Wir analysierten PCS- und Rheuma-typische Beschwerden. Zudem erfolgten umfassende Laboranalysen.

**Ergebnisse:**

Bei 6,25 % (*n* = 5) der PCS-PatientInnen bestand Verdacht auf ERE oder konnte eine ERE gesichert werden. Auch bei den *n* = 75 PCS-PatientInnen ohne ERE zeigte sich ein hoher Überschneidungsgrad von PCS- und rheumatischen Beschwerden. Die Entzündungsparameter CRP und BSG waren bei PCS-PatientInnen mit Verdacht auf eine ERE im Vergleich zu anderen PCS-Betroffenen signifikant höher und waren bei PCS-PatientInnen mit Verdacht auf ERE signifikant häufiger oberhalb des Normbereichs.

**Schlussfolgerung:**

Unsere Arbeit illustriert den hohen Überschneidungsgrad von PCS- mit Rheumasymptomen, ohne dass sich ein weiterführender Verdacht auf eine ERE ergibt. Das Risiko für ERE könnte bei PCS erhöht sein, ein PCS ohne zusätzliche Risikofaktoren, wie z. B. erhöhtes CRP oder Arthritis, rechtfertigt jedoch aus Sicht der AutorInnen keine generelle Vorstellung bei einer RheumatologIn in der klinischen Routine. Diese Empfehlung sollte in größeren Studien weiter untersucht werden.

**Zusatzmaterial online:**

Die Online-Version dieses Beitrags (10.1007/s00393-025-01735-8) enthält Abb. S1, Tab. S1 und S2.

## Abstract (en)

**Background:**

The post-COVID syndrome (PCS) describes long-lasting symptoms after a severe acute respiratory syndrome coronavirus 2 (SARS-CoV-2) infection. PCS and rheumatic diseases, especially collagenoses, show strong overlap of symptoms and biomarkers. Thus far, no biomarkers that differentiate between PCS patients with and without rheumatic diseases exist, and data on the prevalence of rheumatic diseases in this collective in Germany is scarce.

**Method:**

Based on the online platform DEFEAT-Corona, 80 people with PCS without a previously confirmed inflammatory rheumatic disease (IRD) and interest in a rheumatological evaluation were recruited. Typical complaints of PCS and rheumatic diseases were analyzed. In addition, comprehensive laboratory analyses were conducted.

**Results:**

In 6.25% (*n* = 5) of the PCS patients,IRD was suspected or confirmed. The remaining 75 PCS patients without IRD also showed a high degree of overlap with regard to complaints typical for rheumatic disease or PCS. The inflammation parameters C‑reactive protein (CRP) and erythrocyte sedimentation rate (ESR) were significantly higher in PCS patients with suspected IRD compared to patients with PCS only and significantly more often exceeded normal range.

**Conclusion:**

This study illustrates the high degree of overlap between PCS and rheumatic symptoms in PCS patients without previous suspicion of IRD. The risk for IRD could be elevated in PCS. However, in the view of the authors, PCS without additional risk factors, such as elevated CRP or arthritis, does not generally justify rheumatological evaluation in clinical routine. This recommendation should be further investigated in larger studies.

Das Long‑/Post-COVID-Syndrom (PCS) bezeichnet lang anhaltende Symptome nach einer COronaVIrus Disease 2019 (COVID-19)-Erkrankung und betrifft 3–10 % der PatientInnen nach „Severe acute respiratory syndrome coronavirus type 2“(SARS-CoV-2)-Infektionen [1, 2]. Typische Beschwerden sind neuropsychologische Symptome wie prolongierte Erschöpfung (Fatigue) und Konzentrationsschwierigkeiten, welche die Lebensqualität und Teilhabe an Alltagsbetätigungen stark einschränken [3–7]. Aber auch das gehäufte Auftreten chronisch entzündlicher und rheumatischer Erkrankungen wie rheumatoide Arthritis wird bei PCS beobachtet [8, 9]. Immunologische Analysen zeigen eine erhöhte Rate an Markern für chronische Inflammation und Autoantikörpern bei COVID-19 [10], was zur Theorie einer fehlgeleiteten (Auto‑)Immunantwort nach COVID-19 führt [9]. Andererseits ist das Vorliegen von Autoantikörpern wiederum ein Risikofaktor für PCS [11].

Die Versorgungslage für PatientInnen mit PCS ist unzureichend: Bislang gibt es keine kausalen Therapien, und die Behandlungsressourcen in Deutschland sind, ebenso wie für PatientInnen mit entzündlich rheumatischen Erkrankungen, unzureichend [12, 13]. PCS-PatientInnen mit und ohne rheumatische Beschwerden sehen sich mit zum Teil erheblich hohen Wartezeiten konfrontiert, die außerhalb von Früh- und Screening-Sprechstundenmodellen oft Monate betragen können [12, 14].

Aufgrund der Neuheit des Krankheitsbildes existiert bislang noch keine umfassende Analyse rheumatischer Beschwerden bei PCS-PatientInnen in Deutschland. Zudem existieren bislang nur wenige Studien zu klinischen Faktoren und Biomarkern, die PCS-PatientInnen mit einer hohen Wahrscheinlichkeit für das Vorliegen einer rheumatischen Erkrankung von anderen unterscheiden können.

Ziel der vorliegenden Arbeit war es, rheumatische Symptome und Neuerkrankungen von entzündlich rheumatischen Erkrankungen in einer deutschen PCS-Kohorte zu beschreiben. Es erfolgten eine fachärztlich rheumatologische Untersuchung und eine rheumatologische Laboranalyse. Konkret wurden PCS-PatientInnen, die an der online-basierten DEFEAT-Corona-Studie [6, 15] teilnahmen, mittels Anamnese, klinischer Untersuchung und Laboranalysen umfassend fachärztlich rheumatologisch evaluiert.

## Methoden

### Studienpopulation

Diese Untersuchung wurde basierend auf der DEFEAT-Corona-Studie durchgeführt, deren detailliertes Studienprotokoll an anderer Stelle publiziert wurde [16]. Rekrutiert wurden *n* = 104 Personen nach folgenden Einschlusskriterien: (1) Alter ≥ 16 Jahre, (2) persistierende, PCS-typische Symptome ≥ 4 Wochen nach PCR/Antigentest bestätigte SARS-CoV-2-Infektion, (3) Einwilligung zur Studienteilnahme. Die Rekrutierung der Teilnehmenden erfolgte über die Online-Plattform, wobei PCS-PatientInnen mit Interesse an einer Vorstellung in einer rheumatologischen Praxis zur Teilnahme aufgefordert wurden. Der Aufruf (Abb. S1) wurde auf der DEFEAT-Website veröffentlicht und im Mai 2022 an alle Teilnehmenden der DEFEAT-Studie gesandt. Vom 01.06.22 bis zum 23.12.23 wurden in einer rheumatologischen Facharztpraxis in Hannover, Niedersachsen, wöchentlich insgesamt 12 Termine von jeweils 30 min für TeilnehmerInnen des DEFEAT-Corona-Projektes angeboten. Die Vergabe erfolgte ausschließlich online. Jeweils 14 Tage im Voraus wurden die möglichen Termine online gestellt und nach dem Windhundverfahren vergeben.

## Rheumatologische und weitere Symptomevaluation

Bei jeder PatientIn erfolgten eine Anamneseerhebung inklusive eines rheumatologischen Fragebogens zur gegenwärtigen Symptomausprägung und eines standardisierten Fragebogens zu kognitiven Einschränkungen, eine klinische Untersuchung, nach Bedarf eine Gelenksonographie sowie eine Blutentnahme. Fatigue und Lebensqualität wurden mittels standardisierter Werkzeuge wie dem EQ-5D-3L-Index erhoben [17]. Symptomprävalenzen (wie in Tab. 2 präsentiert) wurden mittels einer Likert-Skala gemessen, wobei die Stärken mit 0 (keine) und 10 (stärkste) Symptome angegeben werden konnten. Die Verdachtsdiagnose einer entzündlich rheumatischen Erkrankung wurde nach ExpertInnenmeinung analog zu einer regulären Erstvorstellungssprechstunde gestellt. Hierzu wurden objektivierbare Symptome oder Veränderungen, wie beispielsweise eine Arthritis, zugrunde gelegt. Weiterführende Diagnostik (z. B. Bildgebung oder Verlaufskontrollen) erfolgte in ausgewählten Fällen nach Maßgabe der evaluierenden ÄrztIn („standard clinical care“). Die Laboranalysen umfassten unter anderem C‑reaktives Protein (CRP) und Blutsenkungsgeschwindigkeit (BSG) sowie die Bestimmung weiterer immunologischer Laborparameter (antinukleäre Antikörper [ANA], Rheumafaktor [RF], Antikörper gegen citrullinierte Proteine [CCP-Ak], anti-neutrophile zytoplasmatische Antikörper: Myeloperoxidase [MPO], Proteinase 3 [PR3] ELISA, humanes Leukozytenantigen B27 [HLAB27]) (Methoden: BSG: Sedimentation; CRP, RF: Siemens Healthineers (München, Deutschland), Nephelometrie; sonstige Parameter: Euroimmun (Lübeck, Deutschland): ANA: indirekte Immunfluoreszenz, HLA-B27: EUROArray HLA-B27 Direct, Sonstige: ELISA).

Außerdem erfolgte eine weitere Diagnostik nach Maßgabe der behandelnden RheumatologIn, z. B. wurden insgesamt 13 ENA-Screentests durchgeführt, die allesamt negativ waren. Es wurden außerdem bei 5 PatientInnen Myositispanel durchgeführt, die nur in einem Fall einen grenzwertigen RNP-Ak ergaben. Außerdem wurde bei klinischer Verdachtsdiagnose Sjögren-Syndrom z. B. in 4 Fällen Alpha-Fodrin-Antikörper bestimmt, ohne auffällige Befunde. Weitere Tests, die zur Differenzialdiagnostik durchgeführt, aber deren Resultate nicht im Einzelnen im Manuskript aufgeführt werden, umfassten Analysen von CK, Differenzialblutbild, Ferritin, Leber- und Nierenwerten und Infektionsserologien für Chlamydien, Borrelien, Yersinien oder Hepatitis.

## Ethik

Die Zustimmung der Ethikkommissionen aller teilnehmenden Zentren wurde eingeholt (Medizinische Hochschule Hannover #9948_BO_K_2021, Universitätsmedizin Göttingen „15/8/22 Ü“). Zum Schutz und der Wahrung der persönlichen Rechte der Studienteilnehmenden wurden die in der Deklaration von Helsinki beschriebenen Grundsätze beachtet. Die Studie wurde dem Deutschen Register für klinische Studien unter der Nummer DRKS00026007 gemeldet.

## Statistik

Aufgrund der fehlenden Evidenz zur Thematik war eine A‑priori-Berechnung der Stichprobengröße nicht möglich. PatientInnencharakteristika, Vorerkrankungen, Symptomprävalenz und -stärke sowie Laboranalysen werden deskriptiv dargestellt. Gruppenunterschiede bei ordinal und kontinuierlich ausgeprägten Variablen wurden mittels *Mann–Whitney-U-Test* ermittelt. Nominal ausgeprägte Variablen wurden mittels je nach Kategorienanzahl Fisher’s-Exact- oder Fisher-Freeman-Halton-Test auf Unterschiede zwischen den Gruppen getestet. Ein Zusammenhang zwischen der Symptomstärke und den Laborparametern wurde explorativ mittels Korrelationsanalyse untersucht. *p*-Werte < 0,05 wurden als statistisch signifikant angesehen. Alle Berechnungen und Abbildungen wurden mit R (Version 4.2.3) erstellt.

## Ergebnisse

**Abb. 1:**
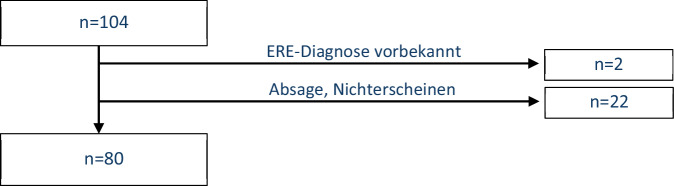
Flussdiagramm zum Ablauf der Rekrutierung

Insgesamt kamen *n* = 104 Teilnehmende der DEFEAT-Corona-Studie dem Aufruf einer Terminbuchung in der rheumatologischen Praxis nach; 21,1 % der Besuche (*n* = 22/104) wurden abgesagt oder nicht wahrgenommen. Bei 2 Personen (2,1 %) bestand bereits vor der COVID-Erkrankung eine entzündlich rheumatische Erkrankung (ERE), sodass diese nicht in die Analyse eingeschlossen wurden. Letztlich wurden *n* = 80 PCS-PatientInnen ohne schon zuvor gesicherte ERE rheumatologisch evaluiert (Abb. 1; Tab. 1).

**Tab. 1:** Demografische und PCS-Krankheitsfaktoren in der Kohorte

	Total (*n* = 80)	PCS ohne ERE (*n* = 75)	PCS mit ERE/V. a. ERE (*n* = 5)	*p*-Wert
*Geschlecht (Missing* *=* *1)*				
Weiblich (*N* =[%])	68 (85 %)	63 (84 %)	5 (100 %)	1,00^1^
*Alter (Median [Q25–Q75]) (Missing 1)*	43,0 (35,5–51,0)	43 (35–51)	44 (43–51)	0,409^2^
*Anzahl sympt. SARS-CoV-2-Infektionen*				
1 (*N* =[%])	58 (72,5 %)	55 (73,3 %)	3 (60 %)	0,612^1^
2 oder mehr (*N* =[%])	22 (27,5 %)	20 (26,7 %)	2 (40 %)	
*Fatigue Assessment Scale (FAS, Missing 1)*				
Median (Q25–Q75)	28,0 (22,0–31:0)	27,5 (21,3–31,0)	28,0 (24,0–30,0)	0,976^2^
Keine Ermüdung	2 (3 %)	2 (27 %)	0 (0 %)	1,00^3^
Ermüdung	26 (32,5 %)	24 (32,4 %)	2 (40 %)	
Extreme Ermüdung	51 (63,8 %)	48 (64,9 %)	3 (60 %)	
*EQ-5D-3L (Median [Q25–Q75])*	0,65 (0,37–0,75)	0,65 (0,37–0,75)	0,39 (0,38–0,65)	0,380^2^

^1^Fisher’s-Exact-Test

^2^Mann-Whitney-U-Test

^3^Fisher-Freeman-Halton-Test

Alle PatientInnen wurden in der rheumatologischen Facharztpraxis im Rahmen einer Anamneseerhebung und Untersuchung fachärztlich evaluiert. Bei 2 PCS-PatientInnen war eine rheumatische Erkrankung bereits nach der COVID-Infektion vermutet worden, die im Rahmen der aktuellen Analysen bestätigt wurde (2,5 %), bei drei weiteren PatientInnen (3,75 %) bestand der Verdacht auf eine bislang nicht bekannte ERE, sodass eine weitergehende Diagnostik empfohlen wurde, bei *n* = 75 PatientInnen bestand kein Verdacht auf eine entzündlich rheumatische Erkrankung.

Konkret zeigte sich bei *n* = 5/80 PCS-PatientInnen (6,25 %) eine ERE mit Arthritis. Bei drei PatientInnen lag eine Mon- und bei zwei weiteren eine Oligoarthritis vor (Tab. 1). Die Tab. 2, 3 und 4 beschreiben die PatientInnenkohorte im Detail und geben Angaben zu Alter, Geschlecht und (Verdachts‑)Diagnosen.

**Tab. 2:** Rheumatologische (Verdachts‑)Diagnosen bei den betroffenen PCS-PatientInnen

Pseudonym	Geschl.	Alter	Diagnose	Arthralgien	Synovitiden	Morgensteifigkeit	Raynaud-Symptomatik	Serositiden
1	W	43	Reaktive Arthritis	Ja	Ja	Nein	Nein	Nein
2	W	37	HLA-B27-neg. Spondyloarthritis mit peripherer Gelenkbeteiligung	Ja	Nein	Nein	Nein	Nein
3	W	44	V. a. Psoriasisarthritis	Ja	Ja	Nein	Möglich	Nein
4	W	51	Undifferenzierte Arthritis	Ja	Ja	Nein	Nein	Nein
5	W	59	V. a. undifferenzierte Arthritis	Ja	Ja	Nein	Nein	Nein

**Tab. 3:** Rheumatologische (Verdachts‑)Diagnosen bei den betroffenen PCS-PatientInnen

Pseudonym	Kutane Symptome	Sicca-Symptom	Fieber	Myalgien	Gastrointestinale Symptome	Augenentzündungen	Lymphknotenschwellungen	Kribbelmiss‑/Taubheitsempfindungen
1	Nein	Nein	Nein	Nein	Nein	Nein	Nein	Nein
2	Nein	Nein	Nein	Ja	Ja	Nein	Nein	Ja
3	Nein	Nein	Nein	Nein	Nein	Nein	Nein	Ja
4	Nein	Ja	Nein	Nein	Nein	Nein	Nein	Ja
5	Nein	Ja	Ja	Ja	Ja	Nein	Nein	Nein

**Tab. 4:** Rheumatologische (Verdachts‑)Diagnosen bei den betroffenen PCS-PatientInnen

Pseudonym	Psoriasis	Steroidsensibilität	Sonstige Beschwerden	Befund Cor	Befund PULMO	Befund Gelenke
1	Kein	Nein	Keine	Unauffällig	Unauffällig	Auffällig
2	Kein	Nein	Keine	Unauffällig	Unauffällig	Unauffällig
3	Ja	Nein	Keine	Unauffällig	Unauffällig	Auffällig
4	Kein	Nein	k. A.	Unauffällig	Unauffällig	Auffällig
5	Ja	Ja	k. A.	Unauffällig	Unauffällig	Auffällig

Der Altersmedian der Kohorte lag bei 43 Jahren und unterschied sich nicht signifikant zwischen den PCS-PatientInnen mit ERE vs. denjenigen ohne. Auch im Hinblick auf das Geschlecht, die Anzahl vorangegangener SARS-CoV-2-Infektionen und wesentliche PCS-assoziierte Symptome wie Grad der Fatigue und krankheitsbezogene Lebensqualität unterschieden sich die beiden PatientInnengruppen nicht (Tab. 1). Auch Komorbiditäten zeigten keine signifikant unterschiedliche Häufigkeit im Vergleich der beiden Gruppen (Tabelle S1 im Anhang).

Die Tab. 5 zeigt Symptome bei PCS-PatientInnen in den ERE vs. Non-ERE-Gruppen. Insgesamt lagen hier keine signifikanten Unterschiede vor. Die häufigsten Symptome bei PCS-PatientInnen mit und ohne Arthritis waren Fatigue und verminderte Belastbarkeit, an denen unabhängig vom Vorliegen einer ERE alle PCS-PatientInnen litten. Konzentrationsschwierigkeiten lagen ebenfalls bei allen PatientInnen mit Verdacht auf rheumatische Erkrankung und bei 96 % derjenigen ohne rheumatische Erkrankung vor.

**Tab. 5:** Symptomprävalenz (*mindestens 1/10 Symptomstärke auf einer Likert-Skala zwischen 0 [keine] und 10 [stärkste] Symptome) sowie Median und IQR der Symptomstärke stratifiziert nach den beiden Gruppen

	PCS ohne ERE (*n* = 75)				PCS mit ERE/V. a. ERE (*n* = 5)				*p*-Wert	
	*n*	%*	Median*	Q25–Q75*	*n*	%*	Median*	Q25–Q75*	Symptom vorhanden bei PCS-PatientInnen ohne vs. mit Rheuma^1^	Symptomstärke bei PCS-PatientInnen ohne vs. mit Rheuma^2^
Fatigue	75	100	8	6–9	5	100	8	7–8	1,00	1,00
Verminderte Belastbarkeit	75	100	8	7–9	5	100	9	5–10	1,00	0,824
Konzentrationsprobleme	72	96,0	7	4–9	5	100	6	5–6	1,00	0,452
Muskelschmerzen	68	90,7	7	5–8	4	80	8	5–8	0,422	0,815
Schlafstörung	68	90,7	6	2,5–8	5	100	6	5–6	1,00	0,182
Kopfschmerzen	65	86,7	4	2–6	5	100	6	6–8	1,00	0,085
Gelenkschmerzen	65	86,7	6	3–8	5	100	6	5–8	1,00	0,651
Depressionen	61	81,3	3	1–6	4	80	4	1–6	0,889	0,889
Palpitationen (Missing = 2)	57	78,1	2	1–4	3	60	6	0–6	0,236	0,427
Kribbeln	58	77,3	4	1–7	4	80	3	3–9	1,00	0,574
Schwindel	58	77,3	2	1–6	3	60	1	0–1	0,588	0,119
Dyspnoe	55	73,3	2	0–5	4	80	3	1–4	1,00	0,976
Angina pectoris (Missing = 1)	48	64,9	2	0–5	3	60	3	0–4	1,00	0,984
Husten (Missing = 1)	48	64,9	2	0–3	2	40	0	0–6	0,351	0,959
Sehstörungen (Missing = 1)	47	63,5	1,5	0–4	4	80	3	2–5	0,651	0,358
SICCA (Missing = 2)	45	61,6	2	0–4	4	80	5	3–5	0,646	0,944
Halsschmerzen (Missing = 1)	45	60,8	1	0–3	2	40	0	0–4	0,390	0,802
Tinnitus (Missing = 1)	44	59,5	1	0–3	3	60	1	0–1	1,00	0,770
Brustschmerzen (Missing = 1)	43	58,1	1	0–4	3	60	2	0–3	1,00	0,967
Angst	41	54,7	1	0–3	2	40	0	0–2	0,658	0,564
Delir (Missing = 1)	39	52,7	1	0–3	2	40	0	0–1	0,668	0,352
Bauchschmerzen (Missing = 1)	39	51,4	1	0–3	3	60	1	0–2	1,00	0,847
Haarausfall (Missing = 2)	35	47,9	0	0–3	3	60	4	0–4	1,00	0,374
Vermehrtes Auftreten von Infekten	35	46,7	0	0–3	4	80	3	3–7	0,195	0,089
Durchfall (Missing = 1)	32	43,2	0	0–2	2	40	0	0–2	1,00	0,973
Geschmacksstörung (Missing = 1)	29	39,2	0	0–2,8	3	60	1	0–2	0,390	0,618
Übelkeit (Missing = 3)	27	37,5	0	0–1,3	2	40	0	0–1	1,00	0,905
Appetitlosigkeit (Missing = 1)	26	33,8	0	0–2	1	20	0	0–0	1,00	0,469
Ausschlag (Missing = 1)	24	31,1	0	0–1	2	40	0	0–0	0,649	0,533
Ohrenschmerzen (Missing = 2)	21	28,8	0	0–1	1	20	0	0–0	1,00	0,728
Fieber (Missing = 1)	15	18,9	0	0–0	0	0	0	0–0	0,579	0,297
COVID-Toes (Missing = 2)	13	17,8	0	0–0	1	20	0	0–0	1,00	0,988

^1^Fisher’s-Exact-Test

^2^Mann–Whitney-U-Test

Auch im Hinblick auf weitere Symptome zeigten sich viele Überschneidungen: Schlafstörungen sowie Kopf- und Gelenkschmerzen wurden von allen PatientInnen in der ERE-Gruppe und von etwa 90 % der nicht an einer ERE leidenden PCS-PatientInnen angegeben. Auch andere Rheuma-typische Beschwerden wie Muskelschmerzen und Sicca-Symptomatik wurden in beiden Gruppen von wesentlich mehr als der Hälfte der PatientInnen angegeben. Haarausfall wurde von 60 % der PCS-PatientInnen und 47,9 % in Gruppe der nicht rheumatisch erkrankten PatientInnen angegeben, wohingegen rezidivierendes Fieber von 20 % der PatientInnen mit PCS ohne rheumatische Erkrankung, aber von keiner der Personen mit PCS und ERE angegeben wurde.

**Abb. 2:**
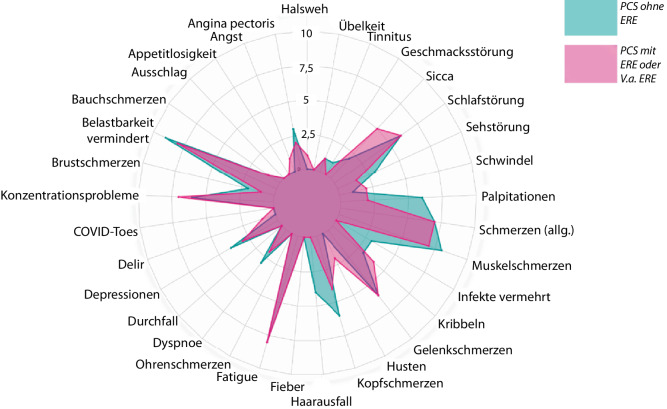
Hohe Überschneidung der Symptomstärke (0 keine Symptome; 10 starke Symptome) zwischen den PatientInnengruppen (PCS-Erkrankte mit rheumatischer Erkrankung oder Verdacht darauf [*lila*] und ohne Rheuma [*türkis*])

Neben der generell starken Überschneidung von Symptomen zeigte sich auch ein hoher Grad der Überlappung in der Symptomstärke zwischen den beiden Gruppen, wie in Abb. 2 illustriert ist (PCS-Erkrankte mit: *lila* und ohne ERE oder V. a. ERE: *türkis*).

**Abb. 3:**
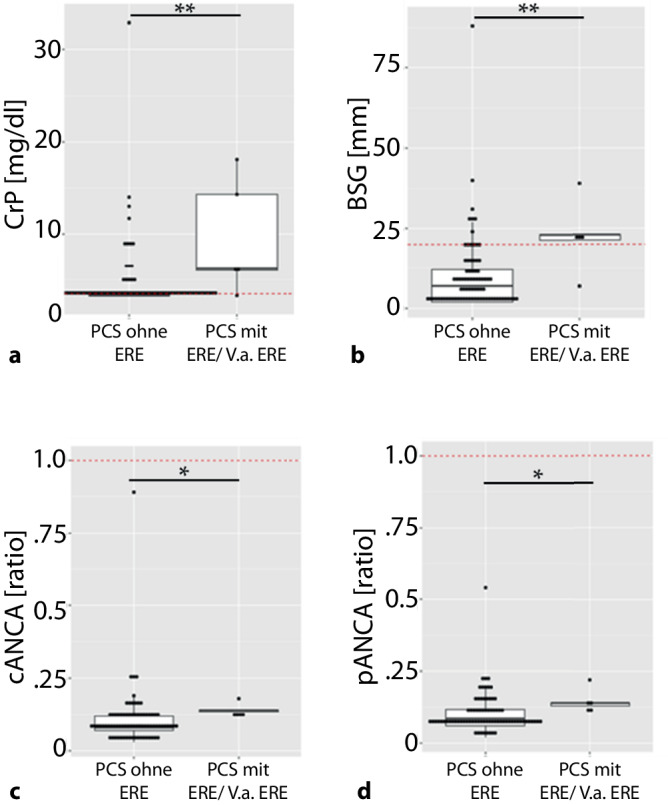
Signifikant höhere Laborparameter bei PCS-PatientInnen mit Verdacht auf entzündlich rheumatische Erkrankung (ERE) als bei solchen ohne. **a** C-reaktives Protein (CRP). **b** Blutsenkungsgeschwindigkeit (BSG). **c**, **d** Anti-neutrophile zytoplasmatische Antikörper (PR3, MRP). Graphen zeigen Median und Q25–Q75, *p < 0,05, **p < 0,01; *rote gestrichelte Linien* stellen dazugehörigen Cut-off-Wert dar

Als Nächstes analysierten wir rheumatologische Laborparameter (ANA, ANCA, BSG, CRP, HLA-B27, RF) in der Kohorte. Die Abb. 3 zeigt die Häufigkeit auffälliger Werte bei den 80 untersuchten PatientInnen.

Die als am häufigsten über dem Referenzwert gemessenen Laborwerte bei den PatientInnen ohne weitere Hinweise auf ERE waren ANA (24 % der Betroffenen) und CRP (14,7 %). Zudem zeigten 9,3 % eine erhöhte BSG. HLA-B27 war in 8 % der Fälle positiv. Dahingegen war in keinem Fall der PCS-PatientInnen mit Verdacht auf rheumatische Erkrankung eine Erhöhung der ANA zu verzeichnen, und bei niemandem in dieser Gruppe bestand der Nachweis von HLA-B27.

Bei den PCS-PatientInnen in der ERE-Gruppe zeigte die überwiegende Mehrheit (80 %) erhöhte BSG- und CRP-Werte. Diese beiden Inflammationsparameter unterschieden sich sowohl in der Frequenz von oberhalb der Norm liegenden Laborwerten als auch im Vergleich der Absolutwerte signifikant zwischen den beiden PatientInnengruppen (Tab. 6 und Abb. 3).

**Tab. 6:** Häufigkeit von auffälligen Laborwerten in beiden Gruppen

		PCS ohne ERE		PCS mit ERE/V. a. ERE		*p*-Wert	
Analyt	Normwert Einheit	Erhöht	Median (Q25–75)	Erhöht	Median (Q25–75)	*p *(Ref)^1^	*p* (Höhe)^2^
ANA	*<* *1:80*	24 (32,0)	–	0 (0,0)	–	0,315	–
RF	*<* *20* *U/ml*	3 (4,0)	10,6 (10,6–10,6)	0 (0,0)	10,6 (10,6–10,6)	1,00	0,370
CCP-AK	*<* *5 (RATIO)*	3 (4,0)	1,0 (1,0–1,1)	0 (0,0)	1,28 (1,0–1,34)	1,00	0,162
MPO (Missing = 2)	*<* *1,0 (RATIO)*	0 (0,0)	0,09 (0,06–0,12)	0 (0,0)	0,14 (0,13–0,14)	1,00	0,025
PR3 (Missing = 2)	*<* *1,0 (RATIO)*	0 (0,0)	0,08 (0,70–0,12)	0 (0,0)	0,14 (0,14–0,14)	1,00	0,019
HLA-B27 (Missing = 2)	*POS/NEG*	6 (8,0)	–	0 (0,0)	–	1,00	–
BSG	*<* *20*	7 (9,3)	6,0 (2,0–11,0)	4 (80,0)	23,0 (21,3–23,0)	0,001	0,006
CRP	*<* *3,5* *mg/dl*	11 (14,7)	3,3 (3,3–3,3)	4 (80,0)	6,2 (6,0–14,3)	0,004	0,005

^1^Fisher’s-Exact Test

^2^Mann–Whitney-U-Test

Die numerischen Werte von PR3 und MPO-Antikörpern waren signifikant höher bei PCS-PatientInnen mit Verdacht auf rheumatische Erkrankung als bei jenen ohne (Tab. 6, Abb. 3a, b), wobei alle gemessenen Werte im Normbereich lagen und somit keine klinische Relevanz haben dürften.

In einer explorativen Korrelationsanalyse der Laborwerte mit jenen Symptomen, die sich zwischen PCS-PatientInnen ohne vs. mit Verdacht auf ERE in ihrer Prävalenz wesentlich unterschieden, zeigte sich eine signifikante, aber allenfalls minimale Korrelation zwischen Haarausfall und der Höhe des RF (r^2^ 0,24, *p* = 0,03), insgesamt bestanden jedoch keine nennenswerten Zusammenhänge zwischen einzelnen Symptomen und Laborparametern (Tabelle S2).

## Diskussion

Wir präsentieren hier erste Daten für die Häufigkeit entzündlich rheumatischer Erkrankungen bei PCS-Erkrankten in Deutschland und zeigen den hohen Grad an Symptomoverlap zwischen diesen beiden Erkrankungen.

Nach konservativen Schätzungen sind in Deutschland mehrere hunderttausend Menschen von PCS betroffen, ohne dass es bislang ein klares Verständnis der Pathomechanismen oder effektive Therapien gibt [12].

Die von uns gefundene Rate an ERE oder Verdacht hierauf lag bei 6,25 %, was darauf hindeutet, dass bei limitierten Ressourcen keine grundsätzliche Indikation zur rheumatologischen Diagnostik bei PCS gegeben sein kann. Der hohe Grad an Symptomüberschneidungen von PCS mit ERE passt jedoch zu Vorarbeiten anderer, die eine chronisch entzündliche Komponente bei PCS und Überschneidung mit ERE-Pathomechanismen beschrieben [9–11]. SARS-CoV‑2 scheint bei entsprechender Suszeptibilität ein Trigger für chronische Inflammation und Autoimmunität zu sein [18]. Für PatientInnen mit PCS wurde ein erhöhtes Risiko für rheumatische Erkrankungen beschrieben [19, 20], und PatientInnen mit rheumatischen Erkrankungen scheinen vice versa ein erhöhtes Risiko für die Entwicklung von PCS zu tragen [21]. Eine Analyse von Versicherungsdaten bei PatientInnen nach COVID-19 ergab in den ersten 3 bis 15 Monaten nach der Erkrankung ein um 40 % erhöhtes Risiko für das Auftreten von RA und/oder Autoimmunerkrankungen [22], auch wenn diese Analyse im Verlauf kontrovers diskutiert wurde [23]. Als mögliche Triggermechanismen für Autoimmunität nach SARS-CoV-2-Infektion wurden unter anderem molekulare Mimikry, Persistenz viraler RNA, Interferon-Dysregulation und neutrophile Inflammation (NETosen) diskutiert [24–26].

Aufgrund der selektierten PatientInnenpopulation (PCS-Betroffene wurden zur Vorstellung in einer rheumatologischen Praxis aufgefordert) kann aus der gefundenen Rate von 6,25 % kein direkter Rückschluss auf die Prävalenz von ERE bei PCS im Allgemeinen gezogen werden. Aus der Literatur sind Zahlen von unter 10 bis hin zu 58 % an bestätigten Rheumadiagnosen bei einer Erstvorstellung in der Rheumatologie bekannt [27, 28], wobei diese hohe Spannbreite insbesondere durch die Definition der Rheumadiagnosen und die vor der Vorstellung eingesetzten Screeningtools (z. B. Apps, Fragebögen, Telefongespräche mit einer rheumatologischen Fachassistenz) erklärt werden kann. Im Vergleich hierzu wäre die in unserer Arbeit gefundene Frequenz von Verdachtsfällen eher gering. In unserer Studie wurde dabei eine konservative Definition des Verdachts auf eine ERE zugrunde gelegt: Es wurden typische Symptome einer ERE ggf. in Kombination mit typischen Laborparametern gefordert.

Unsere Beobachtung einer hohen Rate an Rheuma-typischen Beschwerden bei PCS-PatientInnen mit und ohne Verdacht auf eine ERE ist im Einklang mit Berichten anderer Arbeitsgruppen, die eine ähnlich hohe Rate solcher Symptome für PCS bereits zuvor beschreiben. Karaarslan et al. zeigten, dass ca. 2/5 aller PCS-PatientInnen in den ersten 6 Monaten ihrer Erkrankung mindestens ein Rheuma-typisches, aber unspezifisches Symptom angaben, wobei Fatigue sowie Gelenk- und Muskelschmerzen die am häufigsten genannten Beschwerden waren [29]. Eine Metaanalyse aus dem Jahr 2022, die 54 Studien zum Thema muskuloskeletale Beschwerden nach COVID-19 analysierte, ergab sehr heterogene Befunde z. B. für das Symptom Arthralgie, das mit einer Häufigkeit von 2–65 % der Betroffenen innerhalb eines Zeitraumes von 4 bis 12 Monaten berichtet wurde [30]. In dem von uns untersuchten Kollektiv lagen die Raten – vermutlich aufgrund der Vorselektion (Aufruf zur Vorstellung in einer rheumatologischen Praxis) – höher. Dennoch hatte nur ein geringer Teil dieser PatientInnen sichere Symptome einer ERE.

Im Hinblick auf Symptome oder Biomarker, die zur Identifikation von PCS-PatientInnen mit Verdacht auf Erstmanifestation einer rheumatischen Erkrankung genutzt werden könnten, ließen sich in unserer explorativen Analyse keine hinlänglich spezifischen Merkmale identifizieren, die über bisher bekannte Faktoren wie Gelenkschwellungen und charakteristische anamnestische Angaben hinausgingen. Bei den Laborparametern zeigten sich zwischen den PatientInnengruppen signifikante Unterschiede für CRP, BSG sowie PR3 und MPO. Allerdings waren die beiden letzten Parameter im Schnitt zwar signifikant höher bei PCS-PatientInnen mit Verdacht auf eine rheumatische Erkrankung, lagen jedoch insgesamt bei allen Betroffenen innerhalb des Normbereichs. Vermutlich aufgrund der geringen Anzahl von PCS-PatientInnen mit sicherer oder möglicher ERE in unserer Kohorte konnten keine wegweisenden Assoziationen zwischen einzelnen Symptomen und Laborparametern erfolgen. Der am häufigsten über dem Referenzwert gemessene Laborwert bei Menschen in unserer Kohorte, die keine weiteren Hinweise auf ERE hatten, waren ANA, die jedoch auch bei Menschen ohne chronisch entzündliche Erkrankungen oftmals positiv sind [31].

Zusammenfassend könnten unsere Resultate folgendermaßen interpretiert werden: Bei PatientInnen mit PCS und Verdacht auf eine Rheumaneumanifestation, bei denen klinische Untersuchung und Anamnese zu einer rheumatischen Erkrankung passen, kann ein Basislabor inklusive CRP und BSG hilfreich sein. Wenn diese Parameter erhöht sind und kein anderer Inflammationsfokus besteht, sollte eine rheumatologische Abklärung in Erwägung gezogen werden. Ansonsten ist das Risiko einer entzündlich rheumatischen Erkrankung mit unter 10 % zu niedrig, um ein generelles rheumatologisches Screening zu empfehlen.

Dies ist insbesondere im Bereich der rheumatologischen Frühversorgung wichtig, denn hier bestehen aktuell signifikante Versorgungslücken, die in absehbarer Zeit nicht ohne Weiteres geschlossen werden können [32]. Nur durch die Nutzung strukturierter Frühversorgungsprogramme unter Einbezug von Risikokalkulation, digitalen Evaluationstools und nichtärztlichem Personal kann dem rheumatologischen Versorgungsbedarf adäquat begegnet werden [18, 32, 33].

Unsere Studie ist mit Limitationen behaftet. Wie bereits oben angeführt, war die Gruppe an PCS-PatientInnen mit Verdacht auf ERE klein, sodass hier nur eine explorative Analyse möglich war. Die geringe Fallzahl in unserer Kohorte ist eine wesentliche Limitation unserer Arbeit. Um diese Limitation besser einschätzen zu könne, haben wir eine Post-hoc-Fallkalkulation durchgeführt. Bei *n* = 80 wird die Nullhypothese (Nachweis, dass der Anteil entzündlich rheumatischer Erkrankungen < 20 % liegt) auf dem einseitigen α‑Niveau von 0,05 verworfen, wenn höchstens 9 PatientInnen eine ERE aufweisen. Die Power betrüge 99,9 % bei einer wahren Prävalenz von 3 %, 99,35 % bei einer Prävalenz von 5 und 72,3 % bei 10 %, sodass unsere Fallzahlen durchaus eine akzeptable, wenn auch nicht umfassende Einschätzung der Prävalenzen erlauben. Eine weitere Limitation unseres Ansatzes liegt in einem möglichen Selektionsbias, der durch den Rekrutierungsaufruf, durch den sich v. a. Menschen mit PCS und/oder chronisch entzündlichen Erkrankungen angesprochen gefühlt haben könnten, verursacht worden sein kann. Dies könnte zu einer Überschätzung der Überlappungsfrequenzen beider Erkrankungen geführt haben. Wesentliche Angaben (etwa zu Symptomstärken) wurden zudem von den Teilnehmenden selbst berichtet, was ebenfalls eine Verzerrung der Ergebnisse bedingt haben könnte. Unsere Laboranalytik beschränkte sich zudem auf klassische rheumatologische Autoantikörper sowie nach klinischem Ermessen veranlasste weitere Labordiagnostik, sodass „neue“ PCS-Biomarker oder auch umfangreichere rheumatologische Parameter wie ein routinemäßiges Myositispanel nicht analysiert werden konnten. Zudem sollte beachtet werden, dass aus signifikanten Unterschieden in Serumkonzentrationen von Analyten, deren Werte in der untersuchten Population überwiegend im Normbereich liegen, keine klinischen Konsequenzen gezogen werden sollten.

Weitere Analysen in der DEFEAT-Studie und darüber hinaus zielen auf den Einschluss größerer PatientInnenkohorten und eine Erweiterung der Laboranalysen ab. Auch wurden in dieser Kohorte primär klassische entzündlich rheumatische Erkrankungen mit eindeutiger klinischer Manifestation ausgeschlossen. Dies entspricht dem Auftrag einer rheumatologischen Erstvorstellung in der klinischen Routine. Bei großen Überlappungen der unspezifischen PCS-Symptomatik mit Symptomen entzündlich rheumatischer Erkrankungen könnte ein genaueres Screening, auch mithilfe von z. B. Messung der Ro/SS-A-Antikörper und Speicheldrüsenbiopsien zum Nachweis eines Sjögren-Syndroms bei Siccasymptomatik oder Messung der CK und bei Erhöhung ggf. einem Myositispanel bei Muskelbeschwerden, hier in Zukunft weitere entzündlich rheumatische Erkrankungen aufdecken und bedarf weiterer Forschung.

Zusammenfassend liefern wir hier erste umfassende klinische und laborchemische Daten zu ERE aus einer deutschen PCS-Kohorte. Unsere Arbeit ergab in dieser Kohorte 2 PCS-PatientInnen mit bestehenden und 3 PatientInnen mit neuem Verdacht auf ERE und illustriert den hohen Überschneidungsgrad von PCS mit Rheumasymptomen. Allerdings rechtfertigt diese Prävalenz zum jetzigen Zeitpunkt aus Sicht der AutorInnen keine routinemäßige Vorstellung aller PatientInnen mit PCS bei RheumatologInnen.

Wir hoffen, dass diese Daten dazu beitragen können, die Versorgung von PCS-PatientInnen mit Rheuma in Zukunft zu verbessern.

## Supplementary Information

Abb. S1: Aufruf zur Teilnahme an der Untersuchung, der im Mai 2022 an Teilnehmende der DEFEAT-Studie über E‑Mail versandt wurde und der auf der DEFEAT-Website (nach Login von Teilnehmenden) erschien;
Tab. S1: Komorbiditäten in den beiden PatientInnengruppen;
Tab. S2: Korrelation von Symptomen mit Laborparametern

## Data Availability

Die dem Artikel zugrunde liegenden Daten können nach entsprechender Begründung durch die AutorInnen zur Verfügung gestellt werden, hierzu sollte der korrespondierende Autor kontaktiert werden.
