# Supplementary material for: Inflammatory rheumatic diseases in patients with post-COVID syndrome
Source: Z Rheumatol. 2025 Oct 14;85(3):188–99. [Article in German] doi: 10.1007/s00393-025-01735-8 (PMC13021862; doi:10.1007/s00393-025-01735-8)
Supplement: Supplementary file 1 — Abb. S1: Aufruf zur Teilnahme an der Untersuchung, der im Mai 2022 an Teilnehmende der DEFEAT-Studie über E‑Mail versandt wurde und der auf der DEFEAT-Website (nach Login von Teilnehmenden) erschien; Tab. S1: Komorbiditäten in den beiden PatientInnengruppen; Tab. S2: Korrelation von Symptomen mit Laborparametern [file 393_2025_1735_MOESM1_ESM.pdf]

Anhang

Neu: Modulfragebogen Rheumatologie und Untersuchungsambulanz Rheuma

Einiges deutet darauf hin, dass Long COVID Ähnlichkeiten zu rheumatologischen Erkrankungen haben könnte. Deshalb starten wir nun einen Modulfragebogen zum Thema Rheuma und Corona. Außerdem gibt es für Long COVID Betroffene am Ende die Möglichkeit, an einer Untersuchungsambulanz in unserer Kooperationspraxis teilzunehmen.

Für wen ist der Fragebogen?

Alle unsere Teilnehmer:innen können diesen Fragebogen ausfüllen. Wir wollen herausfinden, ob Menschen mit Langzeitfolgen von Corona rheuma-ähnliche Beschwerden haben. Dafür brauchen wir aber auch eine Vergleichsgruppe. Deshalb freuen wir uns, wenn Sie teilnehmen:

- egal ob Sie Corona hatten oder nicht

- egal ob Sie Langzeitbeschwerden haben oder nicht

Was erwartet mich im Fragebogen?

Es werden Beschwerden abgefragt, die typischerweise bei rheumatischen Erkrankungen vorkommen. Außerdem fragen wir nochmal nach neuen Coronainfektionen und der aktuellen Gesundheitssituation im Allgemeinen.

Modulfragebogen Rheuma

Abbildung S1: Aufruf zur Teilnahme an der Untersuchung, der im Mai 2022 an Teilnehmende der DEFEAT-Studie über Email versandt wurde und der auf der DEFEAT Website (nach Login von Teilnehmenden) erschien. Der rote Button (Modulfragebogen Rheuma) führte zu einem Basisfragebogen, der initiale Daten der ProbandInnen (Alter, Geschlecht, PCS ja/nein) erhob und zum Terminvergabetool führte.

	PCS MIT ERE/V.A. ERE (N=5)	PCS OHNE ERE (N=75)	P-WERT
**ADIPOSITAS**	7 (9,5)	3 (60,0)	0,013
**HYPERTENSION**	8 (10,8)	0 (0,0)	1,00
**NEURODERMITIS**	3 (4,1)	0 (0,0)	1,00
**ALLERGIE**	26 (35,1)	1 (20,0)	0,66
**ASTHMA**	8 (10,8)	0 (0,0)	1,00
**CED**	2 (2,7)	0 (0,0)	1,00
**COPD**	0 (0,0)	0 (0,0)	1,00
**DEMENZ**	0 (0,0)	0 (0,0)	1,00
**DEPRESSIONEN**	8 (10,8)	0 (0,0)	1,00
**DIABETES TYP 1**	2 (2,7)	0 (0,0)	1,00
**DIABETES TYP 2**	0 (0,00)	1 (20,0)	0,063
**EPILEPSIE**	1 (1,4)	0 (0,0)	1,00
**GALLENERKRANKUNG**	3 (4,1)	0 (0,0)	1,00

**GICHT**	0 (0,0)	0 (0,0)	1,00
**HEPATITIS**	0 (0,0)	0 (0,0)	1,00
**HERZINSUFFIZIENZ**	1 (1,4)	0 (0,0)	1,00
**HIV**	0 (0,0)	0 (0,0)	1,00
**HRST**	1 (1,4)	0 (0,0)	1,00
**KHK**	0 (0,0)	0 (0,0)	1,00
**KREBS**	2 (2,7)	0 (0,0)	1,00
**MIGRÄNE**	12 (16,2)	0 (0,0)	1,00
**NIERENINSUFFIZIENZ**	2(2,7)	1 (20,0)	0,180
**SCHMERZEN**	5 (6,8)	0 (0,0)	1,00
**PARKINSON**	0 (0,0)	0 (0,0)	1,00
**PAVK**	0 (0,0)	0 (0,0)	1,00
**PMR**	0 (0,0)	0 (0,0)	1,00
**PSORIASIS**	0 (0,0)	1 (20,0)	0,063
**WUNDEN**	0 (0,0)	0 (0,0)	1,00
**VORHOFFLIMMERN**	0 (0,0)	0 (0,0)	1,00
**SCHILDDRÜSENERKR.**	14	2 (40,0)	0,265

Tabelle S1: Komorbiditäten in den beiden PatientInnengruppen

	**CRP**	**RF**	**CCP-AK**	**ANA**
**FATIGUE**	0,09 (0,42)	-0,18 (0,12)	-0,12 (0,30)	-0,07 (0,53)
**HUSTEN**	0,08 (0,52,)	0,07 (0,56)	-0,02 (0,88)	-0,15 (0,19)
**MUSKELSCHMERZ.**	0,09 (0,44)	0,08 (0,50)	-0,09 (0,45)	-0,06 (0,58)
**SICCA**	-0,04 (0,76)	-0,17 (0,14)	0,01 (0,91)	0,04 (0,71)
**PALPITATIONEN**	0,06 (0,62)	0,11 (0,35)	-0,01 (0,92)	0,09 (0,44)
**HAARAUSFALL**	0,17 (0,13)	0,24 (0,03))	-0,06 (0,58)	0,06 (0,58)

Tabelle S2: Korrelation von Symptomen mit Laborparametern. Angegeben ist jeweils sind Korrelationskoeffizient und in Klammern der dazugehörige p-Wert
